# Phenotypic Traits and Immunomodulatory Properties of *Leuconostoc carnosum* Isolated From Meat Products

**DOI:** 10.3389/fmicb.2021.730827

**Published:** 2021-08-25

**Authors:** Stefano Raimondi, Gloria Spampinato, Francesco Candeliere, Alberto Amaretti, Paola Brun, Ignazio Castagliuolo, Maddalena Rossi

**Affiliations:** ^1^Department of Life Sciences, University of Modena and Reggio Emilia, Modena, Italy; ^2^Biogest-Siteia, University of Modena and Reggio Emilia, Modena, Italy; ^3^Department of Molecular Medicine, University of Padova, Padova, Italy

**Keywords:** *Leuconostoc carnosum*, growth kinetic, substrate preference, antibiotic resistance, biofilm, exopolysaccharide, immunomodulation

## Abstract

Twelve strains of *Leuconostoc carnosum* from meat products were investigated in terms of biochemical, physiological, and functional properties. The spectrum of sugars fermented by *L. carnosum* strains was limited to few mono- and disaccharides, consistently with the natural habitats of the species, including meat and fermented vegetables. The strains were able to grow from 4 to 37°C with an optimum of approximately 32.5°C. The ability to grow at temperatures compatible with refrigeration and in presence of up to 60 g/L NaCl explains the high loads of *L. carnosum* frequently described in many meat-based products. Six strains produced exopolysaccharides, causing a ropy phenotype of colonies, according to the potential involvement on *L. carnosum* in the appearance of slime in packed meat products. On the other side, the study provides evidence of a potential protective role of *L. carnosum* WC0321 and *L. carnosum* WC0323 against *Listeria monocytogenes*, consistently with the presence in these strains of the genes encoding leucocin B. Some meat-based products intended to be consumed without cooking may harbor up to 10^8^ CFU/g of *L. carnosum*; therefore, we investigated the potential impact of this load on health. No strains survived the treatment with simulated gastric juice. Three selected strains were challenged for the capability to colonize a mouse model and their immunomodulatory properties were investigated. The strains did not colonize the intestine of mice during 10 days of daily dietary administration. Intriguingly, despite the loss of viability during the gastrointestinal transit, the strains exhibited different immunomodulatory effect on the maturation of dendritic cells *in vivo*, the extent of which correlated to the production of exopolysaccharides. The ability to stimulate the mucosal associated immune system in such probiotic-like manner, the general absence of antibiotic resistance genes, and the lack of the biosynthetic pathways for biogenic amines should reassure on the safety of this species, with potential for exploitation of selected starters.

## Introduction

The genus *Leuconostoc* encompasses heterofermentative Lactobacillales sharing facultative anaerobiosis, intrinsic vancomycin resistance, catalase negativity, ovococcoid morphology, and dextran production (Björkroth et al., 2014). Carbohydrates are catabolized through pentose phosphate and phosphoketolase pathway, yielding lactic acid, CO_2_, and ethanol and/or acetic acid (Candeliere et al., 2021). The ratio between the amounts of ethanol and acetic acid depends on the ability of the microorganism to re-oxidize the NADH generated in the early stages of the process along with the energy requirements.

*Leuconostoc carnosum* frequently occurs in the microbiota of foods, mostly meat-based products (Shaw and Harding, 1989; Samelis et al., 2000; Goto et al., 2004; Raimondi et al., 2018; Raimondi et al., 2019), but bacteria belonging to this species have been isolated from processed vegetables as well (Hong et al., 2014; Jung et al., 2014). It is highly adapted to grow in meat environments where it can outcompete undesired microorganisms and reach high levels of viable bacteria (Raimondi et al., 2018, 2019). For instance, *L. carnosum* tend to dominate the microbiota of vacuum-packaged or modified atmosphere packaged (MAP) meat products, appearing in the first days after packaging and reaching remarkably high loads (in the order of 10^8^ CFU/g) toward the end of the shelf life, when they dominate the microbiota (Björkroth et al., 1998; Vasilopoulos et al., 2010; Raimondi et al., 2019). Other meat products colonized by *L. carnosum* are sausages, vacuum-packaged smoked bacon, and sliced cooked poultry (Geeraerts et al., 2018; Li et al., 2019).

The high load of *Leuconostoc carnosum* in several foods emphasizes the need of a deeper investigation of this species, taking also into account the controversial role in spoilage and in biopreservation. Indeed, *L. carnosum* has been claimed as responsible of meat deterioration by souring, discoloration, gas production, and slime formation (Shaw and Harding, 1989; Björkroth et al., 1998; Samelis et al., 2006; Raimondi et al., 2019). Likewise other lactic acid bacteria (LAB) in food, members of the genus *Leucosnostoc* take part to a wide range of metabolic processes affecting the taste, flavor, and sensorial properties, such as the fermentation of carbohydrates into organic acids, the metabolism of amino acids, citrate, polysaccharides, polyols, and aldehydes, and the hydrolysis of glycosides, proteins, and lipids (Hemme and Foucaud-Scheunemann, 2004; Ammor and Mayo, 2007; Morandi et al., 2013). Moreover, the release of organic acids and hydrogen peroxide, which exert an intrinsic antimicrobial effect, and production of bacteriocins concur to food preservation and has been well documented in LAB and *Leuconostoc* (Ammor and Mayo, 2007; Morandi et al., 2013).

Based on genomic annotation, most of the strains of *Leuconostoc carnosum* have the genes for bacteriocin synthesis, secretion, and immunity, including the production of leucocin B, a class IIc bacteriocin effective against *L. monocytogenes* (Felix et al., 1994; Candeliere et al., 2021). Comparative genomics revealed that *L. carnosum* consist of a compact group of closely related bacteria sharing most of the metabolic features (Candeliere et al., 2021). Adaptation to a nitrogen-rich environment such as meat is consistent with a number of peptidase genes in the core genome and by the auxotrophy for nitrogen compounds including some amino acids, vitamins, and cofactors. Interestingly, *L. carnosum* genome does not harbor genes for biogenic amines production nor genetic determinants for antibiotic resistances. Although genomics of *L. carnosum* have been investigated, the metabolic activities of this species can be only partially deduced from the genome and additional biochemical efforts are required to evaluate the phenotypic potential of strains that possibly impact on sensorial features of foods and that can present potential industrial interest. Moreover, information on the potential effect of this species on health is still lacking, especially considering that remarkably high counts of live *L. carnosum* are ingested with the consumption of certain ready-to-use meat-based foods.

In this study, the phenotypic diversity of 12 strains of *Leuconostoc carnosum* was thoroughly investigated, including metabolic, technological, and health-related properties. All the tested strains have been genome sequenced (BioProject accession number PRJNA542256) and annotated and were isolated from MAP cooked ham or from fresh sausages (Candeliere et al., 2020, 2021). *In vitro* experiments aimed to assess the role of *L. carnosum* in food spoilage and/or biopreservation. The growth kinetics at different temperatures, the resistance to oxidative, and osmotic stress, the substrate preferences, the proteolytic, and lipolytic capabilities, the ability to yield biogenic amines, and exopolysaccharides, the susceptibility to antibiotics, and the production of anti-*Listeria* bacteriocins were investigated. For the first time, an animal trial was carried out to assess whether *L. carnosum* has potential to reach and colonize the intestine of mice and to exert some effect on the immune system.

## Materials and Methods

### Strains, Media, and Culture Conditions

All chemicals were purchased from Sigma-Aldrich (St. Louis, MO, United States) unless otherwise stated. The strains of *Leuconostoc carnosum*, all originating from MAP sausage or cooked ham (Raimondi et al., 2018, 2019), were obtained from our laboratory collection. The strains were routinely cultured for 48 h at 30°C in static tubes of deMan, Rogosa, and Sharpe broth (Lactobacilli MRS Broth; BD Difco, Sparks, MD, United States), hereinafter referred to as MRS.

Cultures were incubated at 4, 8, 15, 23, 30, 37, and 42°C to determine the effect of temperature on growth kinetic and yield. Turbidity at 600 nm (OD_600_) was used to evaluate growth. OD_600_ data from the exponential tract of the growth curve were used to calculate the specific growth rate (μ_max_). Optimum, minimal, and maximal growth temperature (Topt, Tmin, and Tmax) were deduced with the Sym’Previus software^1^ fitting in a secondary growth model the μ_max_ data as a function of temperature (Rosso et al., 1993).

### Tolerance to Osmotic and Oxidative Stress

Tolerance of *Leuconostoc carnosum* strains to osmotic and oxidative stress was tested in MRS broth supplemented with increasing concentrations of NaCl (0, 20, 40, 60, 80, and 100 g/L) and H_2_O_2_ (0, 0.0625, 0.125, 0.25, 0.5, and 0.75 g/L), respectively (Montanari et al., 2018; Fessard and Remize, 2019). The cultures were seeded (5% v/v) with 24-h MRS cultures and incubated at 30°C for 48 h, then the OD_600_ was measured.

### Biochemical Characterization

The strains were tested for the fermentation of 49 carbohydrates using API 50 CH test strips (bioMerieux, Marcy-l’Etoile, France), according to the manufacturer’s instructions. Briefly, the bacterial biomass from the surface of MRS-agar plates was used to create a suspension with a turbidity equivalent to 2 McFarland in 10 ml of API 50 CHL Medium. The suspension was used to inoculate each tube of the strip. Anaerobiosis was obtained in the inoculated cupules by using sterile paraffin oil. The results were read after 48 h of incubation at 30°C.

A modified formulation of MRS, where glucose was replaced by 5 g/L arginine (hereinafter referred to as MRS-arg), was used to evaluate arginine utilization. MRS-arg cultures, firstly seeded (5% v/v) with 24-h MRS cultures, were incubated for 24 h and propagated six times in the same medium. Then, OD_600_ was measured and compared with that achieved by control MRS cultures.

Citrate fermentation was evaluated in citrate medium (Milliere et al., 1989), containing the following: 4.5 g/L citric acid, 10 g/L proteose peptone (no. 3; BD Difco), 5 g/L yeast extract, 2 g/L K_2_HPO_4_, 5 g/L sodium acetate ⋅ 3H_2_O, 1 g/L Tween 80, and 0.2 g/L MgSO_4_⋅7H_2_O. Cultures in citrate medium, firstly seeded (5% v/v) with 24-h MRS cultures, were incubated for 48 h and propagated six times in the same medium. Then, OD_600_ was measured and compared with that achieved by control cultures containing glucose.

### Proteolytic and Lipolytic Activity

Proteolytic activity was assayed in MRS-agar plates supplemented with 30 g/L gelatin (BD Difco) (Smith and Goodner, 1958). Spots of 5 μl of grown cultures of *Leuconostoc carnosum* were deposited onto the surface of the plates. After 72 h of incubation at 30°C, the plates were placed at 4°C for 5 h, then the diameter of the hydrolysis halo around the spots was measured.

Lipolytic activity was assayed in a MRS-agar medium containing triglyceride emulsions. It was composed of 900 ml of MRS, supplemented with 20 ml of 1 g/L rhodamine solution and an emulsion of 50 ml ddH_2_O, 30 ml of seed oil, and 150 μl of Tween 80. Rhodamine solution and the oil emulsion were sterilized separately (i.e., filtered at 0.22 μm and autoclaved, respectively) and mixed to the sterile MRS. Spots of 5 μl of grown cultures of *Leuconostoc carnosum* were deposited onto the surface of the plates. After 72 h of incubation at 30°C, the plates were placed under an UV lamp and the diameter of the hydrolysis halo around the spots was measured.

### Antibiotic Susceptibility

The antibiotic susceptibility of *Leuconostoc carnosum* was assayed according to the EUCAST protocol (Matuschek et al., 2014). The 24-h MRS cultures were diluted in sterile saline to create suspensions with a turbidity equivalent to 0.5 McFarland and spread onto MRS-agar plates, on the surface of which the antibiotic paper disks were applied. The following antibiotics were assayed: amoxicillin/clavulanic acid, AMC (30 μg); ampicillin, AMP (10 μg); azithromycin, AZM (15 μg); bacitracin, BAC (10 IU); cefixime, CFM (5 μg); ciprofloxacin, CIP (5 μg); clarithromycin, CLR (15 μg); kanamycin, KAN (30 μg); neomycin, NEO (30 μg); tetracycline, TET (5 μg); vancomycin, VAN (5 μg). The inhibition halo was measured after 48 h of incubation at 30°C. Strains were assessed as sensitive (≥20 mm), intermediate (15–19 mm), and resistant (≤14 mm), as previously described (Szutowska and Gwiazdowska, 2021). *Escherichia coli* ATCC 2592 was used as reference strain in the disk diffusion test.

### Anti-Listeria Activity and Cross-Immunity

Grown cultures of *Leuconostoc carnosum* strains and the corresponding supernatants were assayed for the ability to inhibit *Listeria monocytogenes* in a plate assay. The supernatants were recovered by centrifugation (5,000 × *g* for 10 min) and split in aliquots, treated with one of the following: (1) no treatment; (2) the pH was adjusted to 6.0 with 1 M NaOH; (3) addition of 0.1 mg/ml catalase, incubation at 25°C for 30 min, then at 80°C for 10 min; (4) addition of 0.2 mg/ml proteinase K, incubation at 37°C for 1 h, then at 65°C for 10 min. The supernatant aliquots were filter-sterilized at 0.22 μm. A lawn of *L. monocytogenes* was streaked on surface of Brain Heart Infusion (BHI) agar plates (BD Difco), then 20 μl of sample (i.e., *L. carnosum* culture or supernatant aliquots) was spotted on the plates. The diameter of the zone of inhibition was measured after 16 h of incubation at 37°C.

For cross-inhibition test, *Leuconostoc carnosum* strains were inoculated at approximately 10^7^ CFU/ml in agarized MRS plates and challenged, by the agar well diffusion method, against 50 μl of filter-sterilized supernatant of each *L. carnosum* MRS culture grown at 30°C for 48 h. The diameter of the inhibition halos was measured after 48 h of incubation at 30°C.

### EPS and Biofilm Production

Exopolysaccharide (EPS) production was deduced from the mucoid aspect of *Leuconostoc carnosum* colonies that originated onto MRS-agar plates supplemented with 40 g/L sucrose, after 14 days of incubation at 4°C or 48 h at 25, 30, and 37°C (Fessard et al., 2016).

Biofilm formation was quantified with crystal violet in a microtiter assay (Landeta et al., 2013). *Leuconostoc carnosum* was inoculated in MRS broth supplemented with 0.1 g/L Tween 80 and incubated for 24 h at 30°C. Grown cultures were 10-fold diluted in the same medium and poured in a 96-well microtiter plate, 150 μl each well. After 14 days of incubation at 4°C or 48 h at 30°C, the supernatants were removed and the biofilms adhering the wells were washed twice with PBS and stained with 125 μl of 1% Crystal Violet dye for 15 min. Stained biofilms were rinsed six times with PBS and extracted with ethanol/acetone solution (8:2 v/v) for 15 min. The absorbance of the extract was measured at 570 nm and normalized by the OD_600_ of the culture. Strains with values >1 were regarded as positive to biofilm formation (Amaretti et al., 2020).

### Tolerance to Simulated Gastric and Intestinal Juices

Simulated gastric juice consisted of 3.2 g/L pepsin, 0.084 M HCl, and 0.03 M NaCl, with the pH adjusted to 1.4 (Vecchione et al., 2018). Simulated intestinal fluid consisted of 8.5 g/L NaCl, 3.0 g/L bile salts (Oxgall, BD Difco), and 1.0 g/L pancreatin, with the pH adjusted to 7.4 (Castro-Rodríguez et al., 2015). Both the gastric and the intestinal fluid were filter sterilized at 0.22 μM.

The biomass of the strains was recovered by centrifugation (13,000 × *g* at 4°C for 5 min) from 1 ml of a 24-h MRS culture and resuspended in sterile saline. The suspension was diluted to 10^6^ CFU/ml in the gastric or the intestinal juice and incubated at 37°C for 1 or 2 h, respectively. Viable counts were determined with MRS-agar plates. To test the resistance to a sequential treatment with simulated gastric and intestinal fluids, the biomass was recovered by centrifugation (13,000 × *g* at 4°C for 5 min) after the gastric incubation, washed in PBS, then resuspended and incubated in simulated intestinal fluid.

### Mice Colonization

All experimental protocols involving mice were approved by the Animal Care and Use Ethics Committee of the University of Padova under license from the Italian Ministry of Health, and they were in compliance with the national and European guidelines for handling and use of experimental animals.

The biomass of three strains (*Leuconostoc carnosum* WC 0318, *L. carnosum* WC 0319, and *L. carnosum* WC 0321) grown for 48 h in MRS broth was harvested by centrifugation, suspended in PBS containing 15% v/v glycerol to obtain single-dose aliquots of 5 × 10^8^ CFU in 150 μl, then stored at −80°C. Aliquots were thawed one by one and used in the animal trial.

Six-week-old Balb/c male mice were obtained from Envigo Laboratories (Oderzo, Italy) and housed in groups of two per individually ventilated cage, at 22 ± 2°C under a 12-h light–dark cycle. Chow food and water were provided *ad libitum*. Mice were allowed to acclimate to the laboratory for 1 week before entering experimentation. Three groups of six mice were established, each group being assigned a *Leuconostoc carnosum* strain. Each mouse received a daily dose of *L. carnosum* biomass via gastric gavage for 10 days. A control group consisted of six mice daily receiving 150 μl of PBS containing 15% v/v glycerol. Fecal pellets were collected at the end of the treatment, then immediately stored at −80°C in pre-weighted tubes containing 50% glycerol. Viable *L. carnosum* in mice feces were enumerated with pour plate method within MRS-agar supplemented with 2 μg/ml vancomycin, 25 μg/ml trimethoprim, and 12.5 μg/ml cycloheximide. The plates were incubated at 30°C for 72 h.

### *In vivo* Effect on Immune System

The mice were sacrificed after 10 days of daily supplementation with *Leuconostoc carnosum* WC 0318, *L. carnosum* WC 0319, *L. carnosum* WC 0321, or PBS. The whole ileum was collected, washed in cold PBS, and the Peyer’s patches were isolated under a dissecting microscope. Mucosa was scraped off from the underlying muscle layer of distal ∼5 cm of ileum and immediately frozen in liquid nitrogen and stored at −80°C.

Peyer’s patches were placed in DMEM containing dithiothreitol for 30 min to remove the mucus and then incubated for 90 min at 37°C in Hanks’ balanced salt solution without calcium but added with EDTA to remove epithelial cells. Peyer’s patches were extensively washed with Hanks’ balanced salt solution without calcium and then digested with collagenase D (2 mg/ml) and DNase (10 μg/ml) for 5 min at 37°C. Mononuclear cells were incubated for 30 min in ice-cold PBS containing 2% BSA or murine serum (blocking buffer) to block unspecific bindings. Cells were washed twice by centrifugation in PBS (1,600 rpm, 6 min) and incubated with the appropriate antibodies (Supplementary Table 1). Finally, cells were washed with blocking buffer and analyzed using a FACSCalibur (Becton Dickinson, San Jose, CA, United States).

Cells were first selected on a forward scatter and side scatter dot plot. CD11c double-positive cells were recorded in 10,000 events. For intrakine analysis, CD4 or CD8 positive lymphocytes were selected on FL-3/SSC dot plot and CD25/Foxp3 or IL17 positive cells were recorded in 50,000 events.

Frozen mucosal specimens were weighed and placed in ice-cold PBS buffer (1:10 w/vol ratio) supplemented with protease inhibitors (0.1 mM PMSF, 1 μM leupeptin, 150 nM aprotinin) and homogenized for 30 s. Cellular debris was removed by centrifugation (10,000 × *g* for 10 min at 4°C). Levels of interleukin (IL)-1β and IL-10 were determined in the cleared supernatants by ELISA, using commercially available kits (eBioscience, Prodotti Gianni, Milano, Italy). The assays were conducted following the manufacturer’s recommended protocols.

### Statistical Analyses

Comparisons were performed with Student’s *t*-test or one-way ANOVA followed by Tukey *post hoc* test. Statistical analysis was done using SPSS Statistics 21 (IBM Corp., Armonk, NY, United States).

Jaccard’s similarity scores among the strains were calculated with the software Past ver. 4.05 (Hammer et al., 2001) considering their metabolic profile, antibiotic susceptibility, and other physiological properties (Supplementary Table 2) and were used to compute an UPGMA (unweighted pair group method with arithmetic mean) cladogram.

## Results

### Growth Kinetics and Temperature Optimum

**FIGURE 1:**
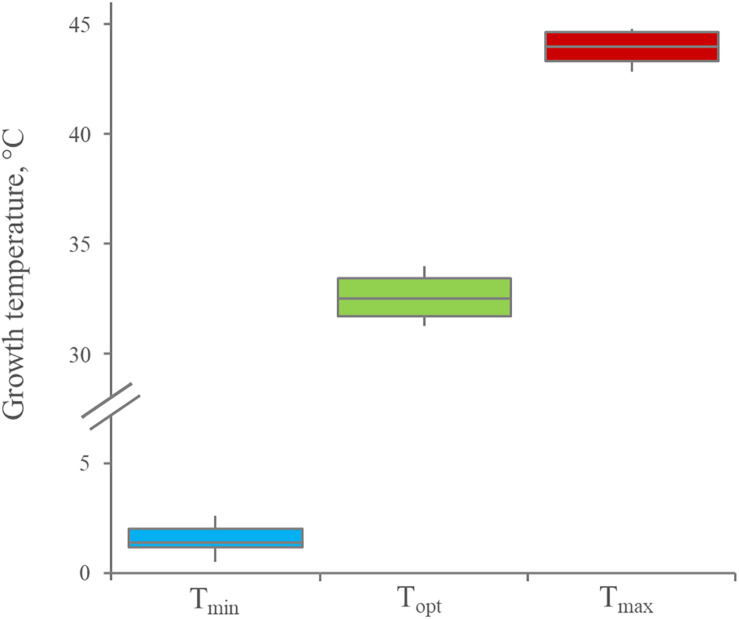
Distribution of minimum, optimum, and maximum growth temperature for 12 *Leuconostoc carnosum* strains, cultured in MRS broth for 48 h. Boxes indicate the 25th, 50th, and 75th percentiles; whiskers indicate the 10th and 90th percentiles.

**FIGURE 2:**
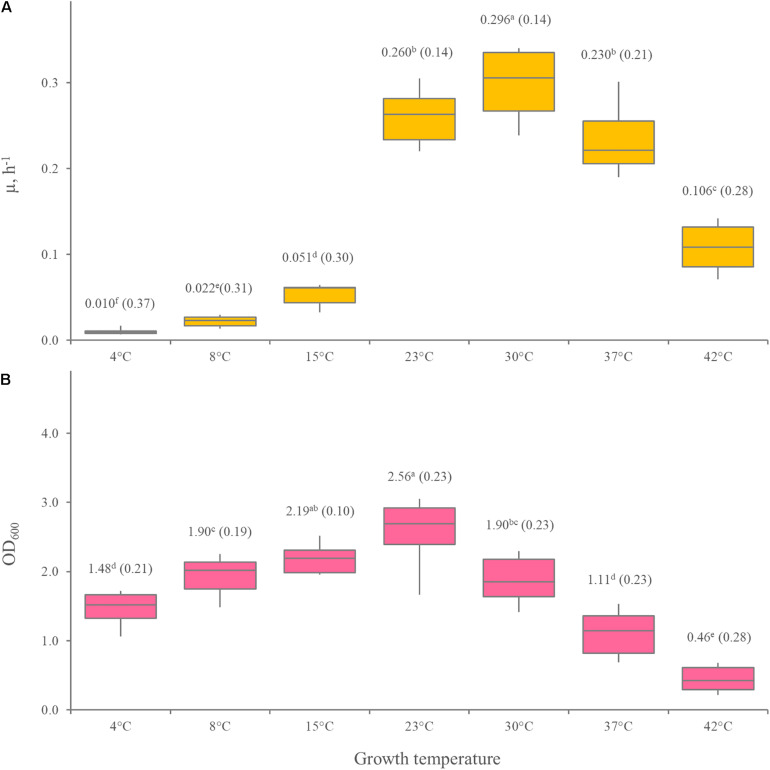
Effect of temperature on μ_max_ **(A)** and the biomass yield **(B)** of 12 *Leuconostoc carnosum* strains, cultured in MRS broth for 48 h. **(B)** Boxes indicate the 25th, 50th, and 75th percentiles; whiskers indicate the 10th and 90th percentiles. Mean values are reported in the labels, with the coefficient of variation in brackets. Within each panel, means with different letters significantly differ (*p* < 0.05, ANOVA with Tukey’s *post hoc*).

The growth kinetics of 12 *Leuconostoc carnosum strains* was studied in MRS broth. All the strains grew in the temperature ranging from 4 to 37°C, with difficulty at 42°C. The optimum temperature was predicted in the range of 30.6–34.2°C (median = 32.5°C), while minimum and maximum ones lay in the range of 0.3–3.0°C (median = 1.4°C) and 42.6–44.8 (median = 44.0°C), respectively, (Figure 1). The maximum specific growth rate (μ_max_) of all the strains was the highest at 30°C (*p* < 0.05), ranging from 0.23 to 0.34 h^–1^ with a median of 0.30 h^–1^ (Figure 2A). For all the strains, the highest biomass yields were achieved at 23°C (*p* < 0.05), laying in the OD_600_ range 1.30–3.39, with a median value of 2.56 (Figure 2B). The medium generally presented a pH between 4 and 5 at the end of the growth phase, regardless of the growth temperature (data not shown).

### Resistance to Oxidative and Osmotic Stress

To evaluate the adaptation to oxidative and osmotic stress, *Leuconostoc carnosum* strains were cultured in MRS broth with increasing concentrations of H_2_O_2_ and NaCl. H_2_O_2_ restricted the biomass yield by at least 1 magnitude (*p* < 0.05) at the concentration of 0.0625 g/L, while higher concentrations completely inhibited growth (Supplementary Figure 1). Differences among the strains were registered. In presence of 0.0625 g/L H_2_O_2_, *L. carnosum* WC319 did not grow, most strains achieved an OD_600_ < 0.2, while *L. carnosum* WC0329 performed better, yielding an OD_600_ of 0.36.

**FIGURE 3:**
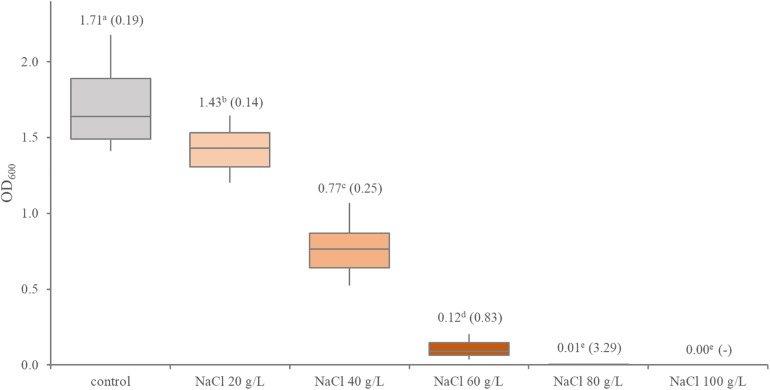
Distribution of the growth performance of 12 *Leuconostoc carnosum* strains in presence of increasing concentrations of NaCl. Boxes indicate the 25th, 50th, and 75th percentiles; whiskers indicate the 10th and 90th percentiles. The labels report the mean and, in brackets, the coefficient of variation; different letter superscripts indicate means that significantly differ (*p* < 0.05, ANOVA with Tukey’s *post hoc*).

In presence of NaCl, *Leuconostoc carnosum* grew with decreasing yields up to 60 g/L (Figure 3). Marked differences were observed among the strains (Supplementary Figure 2). In presence of 60 g/L NaCl, *L. carnosum* WC0327 and *L. carnosum* WC0318 presented the lowest and the highest growth yield, with OD_600_ values of 0.03 and 0.38, respectively. Only *L. carnosum* WC0328 grew to some extent also with 80 g/L NaCl, even though with an OD_600_ < 0.1

### Biochemical Characterization

Substrate utilization by the 12 *Leuconostoc carnosum* strains was assessed with API 50 CH strips and specific growth experiments in modified MRS media (Table 1 and Supplementary Figure 3). All the strains were positive for the utilization of glucose, ribose, sucrose, and esculin. Fructose, mannose, methyl-α-glucopyranoside, N-acetyl-glucosamine, trehalose, and turanose were used by at least 10 strains, while cellobiose, maltose, gentibiose, and gluconate were used by 5–7 strains. On the other hand, all the strains were unable to use 37 substrates, among which glycerol, erythritol, D- and L-arabinose, D- and L-xylose, galactose, L-sorbose, L-rhamnose, inositol, mannitol, sorbitol, lactose, melibiose, inulin, melezitose, raffinose, amidon, glycogen, xylitol, tagatose, fucose, citrate, and arginine. *L. carnosum* WC0329 exhibited the smallest range of growth substrates (6, i.e., glucose, fructose, ribose, sucrose, gluconate, and esculin) and *L. carnosum* WC0323 the largest one (14, i.e., glucose, fructose, ribose, mannose, methyl-α-glucopyranoside, N-acetyl-glucosamine, cellobiose, maltose, esculin, sucrose, trehalose, gentibiose, turanose, and gluconate).

**TABLE 1:** Substrate utilization by the 12 *Leuconostoc carnosum* strains assessed with API 50 CH.

**Substrate**	**No of positive tests (%)**
D-Cellobiose	5 (42)
D-Fructose	10 (83)
D-Glucose	12 (100)
D-Maltose	5 (42)
D-Mannose	10 (83)
D-Ribose	12 (100)
D-Saccharose	12 (100)
D-Trehalose	10 (83)
D-Turanose	11 (92)
Esculin	12 (100)
Gentiobiose	6 (50)
Methyl-α-D-glucopyranoside	11 (92)
N-Acetyl-glucosamine	10 (83)
Potassium gluconate	7 (58)

*Only positive tests were shown, reporting the number and the % of strains able to grow.*

Proteolitic and lipolytic activity were also assessed, showing that all the strains were negative to gelatin and triglyceride hydrolysis.

### Antibiotic Resistance

**FIGURE 4:**
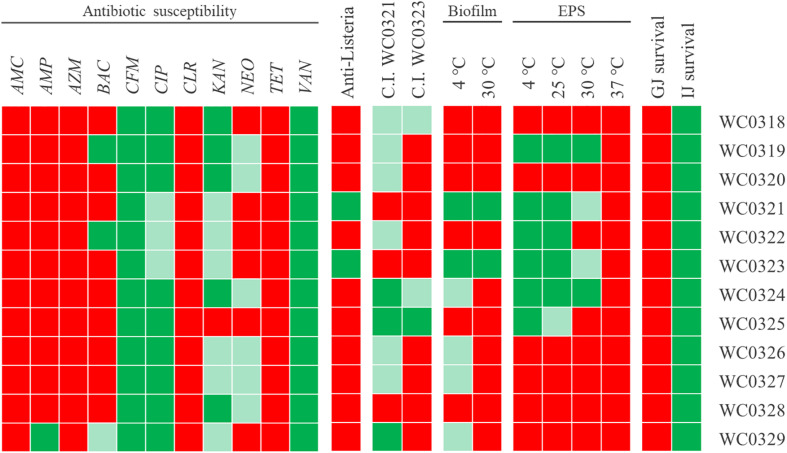
Phenotypic traits of 12 *Leuconostoc carnosum* strains. Resistance (green), susceptibility (red), and intermediate behavior (light green) to amoxicillin/clavulanic acid (AMC), ampicillin (AMP), azithromycin (AZM), bacitracin (BAC), cefixime (CFM), ciprofloxacin (CIP), clarithromycin (CLR), kanamycin (KAN), neomycin (NEO), tetracycline (TET), and vancomycin (VAN); anti*-Listeria* activity and Cross-Immunity test (red, absence of growth inhibition; light green, inhibition zone with diameter <10 mm; green, diameter ≥10 mm); biofilm production (red, OD _570_/OD_600_ < 1; light green, ≥1; green, ≥5); EPS production (red, normal colonies; light green, slightly mucoid colonies; green, mucoid aspect); survival to gastric and intestinal juices (red, absence of viable cells; green, presence of residual growth).

The 12 *Leuconostoc carnosum* strains were screened for susceptibility to 11 antibiotics (Figure 4). All the strains were resistant to cefixime and vancomycin and most of them were resistant or gave an intermediate response to ciprofloxacin and kanamycin. On the other hand, the strains were all susceptible to amoxicillin/clavulanic acid, azithromycin, clarithromycin, and tetracycline and were susceptible or gave an intermediate response to neomycin. Most strains were susceptible to ampicillin and bacitracin, except for a few resistant or intermediate strains. *L. carnosum* WC0329 was resistant to ampicillin, while *L. carnosum* WC0319 and *L. carnosum* WC0322 were resistant to bacitracin.

### Antimicrobial Activity

Only *Leuconostoc carnosum* WC0321 and *L. carnosum* WC0323 inhibited the growth of *L. monocytogenes* to some extent (Figure 4). For both the strains, inhibition of *Listeria* occurred with the culture, the supernatant, the neutralized supernatant, and the supernatant treated with catalase, while the supernatant treated with proteinase K did not exert any inhibition (data not shown).

Growth inhibition by *Leuconostoc carnosum* WC0321 or, to a lesser extent, by *L. carnosum* WC0323 was observed also in the other *L. carnosum* strains, with the exception of *L. carnosum* WC0328 that was immune (Figure 4). *L. carnosum* WC0324 and *L. carnosum* WC0325 were the most sensitive, particularly to the supernatant of *L. carnosum* WC0321.

### EPS and Biofilm Production

EPS production was assayed at 4, 25, 30, and 37°C (Figure 4). The ability to produce EPS was observed in six strains (i.e., *Leuconostoc* WC0319, *L. carnosum* WC0321, *L. carnosum* WC0322, *L. carnosum* WC0323, *L. carnosum* WC0324, and *L. carnosum* WC0325) that presented mucoid colonies when cultured at 4 and 25°C. EPS production tended to reduce with the increase of temperature. A decrease in mucosity was observed in the colonies of *L. carnosum* WC0325 at 25°C and in those of *L. carnosum* WC0321 and *L. carnosum* WC0323 at 30°C. No strains presented mucoid colonies at 37°C.

*Leuconostoc carnosum* WC0321 and *L. carnosum* WC0323 produced considerable biofilm both at 4 and 30°C. A slight biofilm formation was observed only at 4 and not at 30°C for *L. carnosum* WC0324, *L. carnosum* WC0326, *L. carnosum* WC0327, and *L. carnosum* WC0329 (Supplementary Figure 4).

### Resistance to Simulated Gastric and Intestinal Juices

The treatment with simulated gastric juice was lethal for all the strains (Figure 4). On the other hand, all the strains survived to some extent to the treatment with simulated intestinal juice, presenting a residual viability in the range of 16–100% (Figure 4 and Supplementary Table 3). Coherently, no strains survived the sequential incubation, first in the gastric and then in the intestinal juice.

### Hierarchical Clustering of Phenotypic Diversity

**FIGURE 5:**
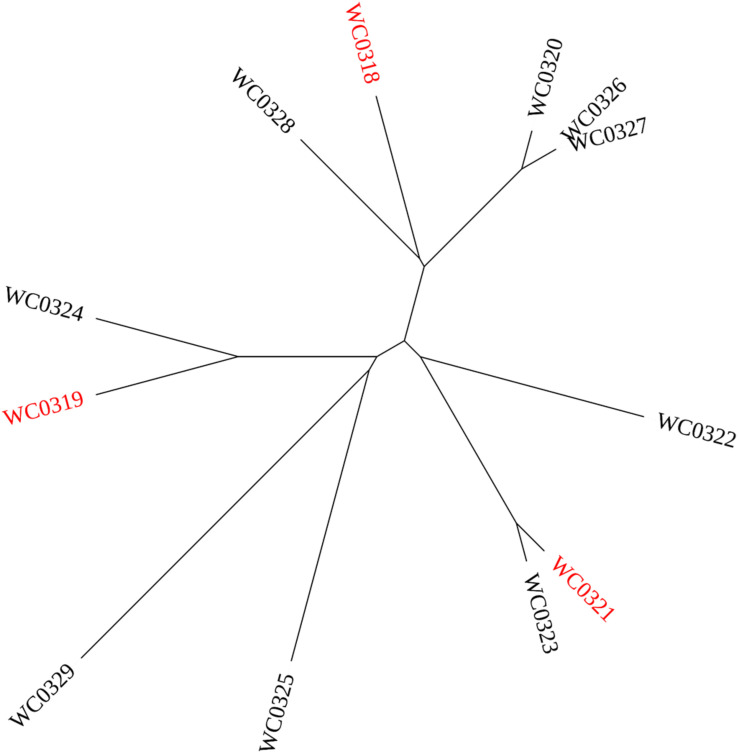
UPGMA cladogram of the 12 strains *Leuconostoc carnosum* according to their phenotypic traits. The strains selected for the mice colonization study are labeled in red.

The hierarchical clustering of the 12 *Leuconostoc carnosum* strains recognized three clades according to Jaccard’s similarity of the phenotypic traits (Figure 5). One clade encompassed *L. carnosum* WC0319, *L. carnosum* WC0324, *L. carnosum* WC0325, and *L. carnosum* WC0329, which were all unable to use the disaccharides cellobiose, maltose, and gentiobiose and were negative to bacteriocin production and biofilm formation, at least at 30°C. The second clade encompassed *L. carnosum* WC0321, *L. carnosum* WC0322, and *L. carnosum* WC0323, which were the most sensitive to kanamycin and neomycin, and produced EPS, with *L. carnosum* WC0321 and *L. carnosum* WC0323 that also exerted anti-*Listeria* activity and were the main producers of biofilm. The third clade, encompassing *L. carnosum* WC0318, *L. carnosum* WC0320, *L. carnosum* WC0326, *L. carnosum* WC0327, and *L. carnosum* WC0328, differed from the first one for the wider spectrum of substrates, particularly the disaccharides.

### Colonization of Mice Gut

The strains *Leuconostoc carnosum* WC0318, *L. carnosum* WC0319, and *L. carnosum* WC0321, each belonging to a different clade, were selected to investigate their potential in colonizing the intestine of mice. They all failed to colonize the intestine of Balb/c mice after 10 days of daily administration at the dose of 5 × 10^8^ cells. For the three mice groups, each receiving a single strain, the viable counts in the fecal samples onto MRS plates did not yield any colony (data not shown).

### Effect on the Intestinal Mucosa-Associated Immune System

**FIGURE 6:**
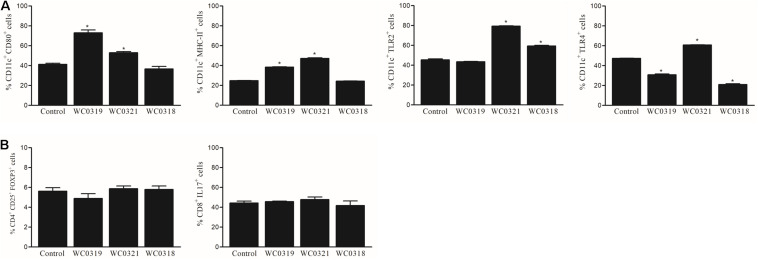
Effect of daily oral supplementation with 5 × 10^8^ CFU of *Leuconostoc carnosum* WC0318, *L. carnosum* WC0319, and *L. carnosum* WC0321 on intestinal mucosa immune cells, determined by FACS: **(A)** DC (CD11c+); **(B)** CD3 + lymphocytes. * Indicates significant difference versus the control (*p* < 0.01, *t*-test).

To evaluate the impact of *Leuconostoc carnosum* on the immune system, the phenotype of dendritic cells (DC) and lymphocytes in Peyer’s patches was analyzed. Following 10 days of supplementation, *L. carnosum* WC0321 enhanced all the measured markers of DC maturation (namely CD-80, MHC-II, TLR2, and TLR4), while *L. carnosum* WC0318 and *L. carnosum* WC0319 had a limited effect (Figure 6). *L. carnosum* effects were restricted to DC, since no effects on polarization of CD3^+^ lymphocytes toward regulatory (CD4^+^ CD25^+^ FoxP3^+^) or inflammatory (CD3^+^ CD8^+^ IL17^+^) phenotype were observed.

**FIGURE 7:**
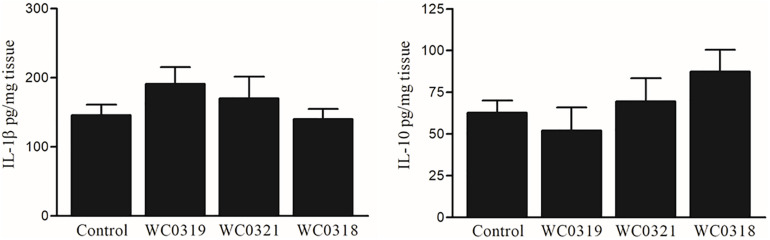
Effect of daily oral supplementation with 5 × 10^8^ CFU of *Leuconostoc carnosum* WC0318, *L. carnosum* WC0319, and *L. carnosum* WC0321 on intestinal mucosa IL-1β and IL-10, quantified by ELISA. Cytokine levels were expressed as pg/ml of tissue lysate. Means did not significantly differ from the control (*p* > 0.05, *t*-test).

To further assess the impact of *Leuconostoc carnosum* on mucosal immune system balance, the expression level of IL-1β and IL-10 was measured after 10 days of supplementation. No significant effects on mucosal cytokine levels were observed (*p* > 0.05), as reported in Figure 7.

## Discussion

Twelve strains of *Leuconostoc carnosum*, the genome of which has been recently subjected to comparative analysis (Candeliere et al., 2021), were investigated in terms of biochemical, physiological, and functional properties. The spectrum of sugars fermented by the strains was limited to few mono- and disaccharides but consistent with the main isolation sources of the species, i.e., meat products and fermented vegetables (Jung et al., 2014; Ishii et al., 2017; Raimondi et al., 2018). All the strains fermented glucose, sucrose, and ribose, coherently with the equipment of genes encoding the transporters for these sugars (i.e., specific phosphotransferase systems for glucose and sucrose, and ABC transporters for ribose) (Candeliere et al., 2021). In meat matrices, glucose derives from the hydrolysis of glycogen, while sucrose is present in plants or in fermented meat products where it may be supplemented to accelerate the fermentation rate. Ribose is abundant in certain vegetables and in meat, where it derives from the ribonucleotides of the muscle (Meinert et al., 2009). Most of the strains (10 out of 12) fermented mannose and/or fructose and were all equipped with PTS transport systems for these sugars which are abundant in fruits and vegetables. All the strains exhibited the ability to ferment trehalose that, mainly in conjugated forms, actively participates in signaling in plants and esculin, a plant glucoside. Even though cellobiose fermentation was restricted to a few strains, in agreement with previous indications (Shaw and Harding, 1989), all the strains harbor the genes for a cellobiose PTS transport system. The utilization of substrates such as trehalose, cellobiose, and esculin suggests a remote and efficient association of *L. carnosum* with plants (Jung et al., 2014; Ishii et al., 2017). Unlike *Latilactobacillus sakei* (former *Lactobacillus sakei*, Zheng et al., 2020) and other LAB that thrive in meat products gaining energy from arginine through the arginine deiminase pathway, *L. carnosum* did not catabolize arginine. Differently from other *Leuconostoc* species, *L. carnosum* was unable to catabolize citrate, coherently with the lack of the genes encoding citrate transporter, citrate lyase, and oxaloacetate decarboxylase, and thus relied only on carbohydrate fermentation for energy supply (Özcan et al., 2019; Candeliere et al., 2021).

As meat products are subjected to treatments or conditions that hamper growth of microorganisms, it is expected that efficient colonizers exhibit some adaptation to cope with hostile factors such as low temperature, pH variations, and osmotic and oxidative stress (D’Angelo et al., 2017). The study of the temperature limits indicated that *Leuconostoc carnosum* is mesophilic, but versatile enough to grow at temperatures that are compatible with refrigeration, thus explaining the bloom in many meat-based products especially throughout long shelf-life periods (Samelis et al., 2000). Furthermore, *L. carnosum* can take advantage of any temperature abuse occurring in refrigerators or over the cold chain since a rise from 4 to 8°C caused a twofold increase of the specific growth rate. The ability of *L. carnosum* to efficiently grow in presence of up to 60 g/L NaCl, although with reduced yields in presence of increasing the amount of salt, is consistent with his adaptability to high-salt food matrices. On the other side, all the strains resulted very sensitive to hydrogen peroxide, according to the absence of catalase.

The increasing demand of healthy, fresh, and natural foods devoid of added chemical preservatives and stabilizers has fostered the search of bioprotective starters, particularly among LAB. Various species belonging to the genus *Leuconostoc*, including *Leuconostoc carnosum*, produce bacteriocin of different classes that target *Listeria* (e.g., leucocins A and C) and other LAB such as *Leuconostoc* and *Weissella* (e.g., leucocin B) (Felix et al., 1994; Parente et al., 1996; Pérez-Sánchez et al., 2011; Wan et al., 2015). This study provides evidence of the protective role of *L. carnosum* WC0321 and *L. carnosum* WC0323 against *L. monocytogenes*, consistently with the presence in these strains of the genes encoding leucocin B and the ABC-like transporter LanT, the former harbored in plasmids and the latter in the genome (Candeliere et al., 2021). Interestingly, these strains exerted antimicrobial activity also against the other *L. carnosum* strains, and it should be investigated if such effect was mediated by the same bacteriocin. Coherently, *L. carnosum* WC0329, which harbors only the plasmid genes for leucocin B but lacks LanT, did not exert any anti-*Listeria* effect. The ability to inhibit other strains and *L. monocytogenes* is encouraging for a possible utilization of selected *L. carnosum* strains as bioprotective starters, to prevent growth of both pathogenic microorganisms and LAB that could participate to spoilage. In particular, bacterial starters that do not produce EPS, biogenic amines, and lipolytic or proteolytic activities could be used to extend the shelf-life of the products, provided that they have no adverse effects on visual, olfactory, and gustative properties (Ammor and Mayo, 2007; Singh et al., 2012; Zarour et al., 2012).

Ready-to-eat meat products may harbor up to 10^8^ CFU/g of *Leuconostoc carnosum*; therefore, the potential impact of this species on health deserves attention. The fact that *L. carnosum* strains did not survive simulated gastric juice reassures on their abatement in the stomach even though they withstood to simulated intestinal fluid. Ingestion of *L. carnosum* is expected not to impact on health, or at least not to determine any intestine colonization, even transient, taking into account the unsuccessful recovery of *L. carnosum* from the feces of mice boosted *L. carnosum*. However, it is still possible that *L. carnosum* is part of the wide and understudied biodiversity of LAB within human feces (Rossi et al., 2016), and its presence within the gut microbiome deserves further investigation.

The dietary supplementation of *Leuconostoc carnosum* strains to mice did not affect the maturation of intestinal lymphocytes and mucosal levels of IL-1β and IL-10 but affected the maturation of surface markers of intestinal DC. The response of DC was higher with *L. carnosum* WC0321, compared with WC0318 and WC0319, confirming that some strains might exert favorable effects on the mucosal associated immune system. The significant difference among the strains is not surprising since strain-specific effects on mucosal immune system is well recognized, particularly in the field of probiotics (Luyer et al., 2005; Kekkonen et al., 2008; McFarland et al., 2018; Sagheddu et al., 2020). Since *L. carnosum* strains do not survive the gastric barrier, their immunomodulatory activity must be ascribed to some bacterial cell components. Indeed, it was demonstrated that capsular structures, cell wall extracts, DNA, and S-layer proteins of LAB can induce the activation of DCs through Toll-like receptor signaling, which play a critical role in the generation of protective immune responses, and that non-viable probiotics could also have significant beneficial effects on human health (Gueimonde et al., 2004; Taverniti and Guglielmetti, 2011; Lahtinen, 2012; Engevik et al., 2021). Intriguingly, *L. carnosum* WC0321 efficiently produced both EPS and biofilm and, unlike *L. carnosum* WC0318 and *L. carnosum* WC0319, harbors genes encoding some tyrosine kinases and phosphatases that regulate the formation of exopolysaccharides, EPS biosynthetic genes of the *ywq* group, and EPS glycosyltransferases. EPS mediate plenty of beneficial properties in probiotic strains and it should be further investigated whether it played some role in inducing the response of DC to *L. carnosum* WC0321 (Angelin and Kavitha, 2020).

On the other side, EPS-producing bacteria participate in deterioration of meat-based foods, such as MAP cooked ham, where they cause the appearance of a ropy slime that is considered unacceptable by consumers for certain products. Six out of 12 strains were positive to the ropy phenotype, with a stronger tendency to form viscous colonies at the lower growth temperatures, and they may be involved in the appearance of slime in food, causing the sensorial quality to decline throughout the shelf life.

Some resistance to antibiotics was highlighted, although it was not predicted by comparative genomics. Vancomycin resistance is a common trait of bacteria belonging to the *Leuconostoc–Weissella* group, ascribable to the structure of the active site of D-alanine–D-alanine ligase (Flórez et al., 2016). In a similar way, the shared resistance to the β-lactam cefixime and to the fluoroquinolone ciprofloxacin may be due to some intrinsic mechanism. On the other hand, the resistance to ampicillin or bacitracin exhibited by few strains can be due to some mechanism that escaped the detection by genome scanning.

Many traits herein investigated should reassure on the safety of *Leuconostoc carnosum* and leave ample scope for exploitation of selected starters in conserved meats, where the species, coping with low temperatures and presence of the salt, is very competitive. Potential candidates for such a technological application are the bacteriocin producers *L. carnosum* WC0321 and *L. carnosum* WC0323. These strains deserve to be challenged in real food matrix, aiming to validate their biopreservative action and to check if they have adverse effects food sensorial properties, for instance in relation to slime formation. A nutraceutical application of *L. carnosum*, apart from the potential as food biopreservative, could not be excluded but does not seem straightforward. In this perspective, the potential immunomodulatory effect of EPS has to be further investigated, to assess if some relevant health-promoting effect could be ascribed to this bacterial component.

## Data Availability Statement

Publicly available datasets were analyzed in this study. This data can be found here: PRJNA542256.

## Ethics Statement

The animal study was reviewed and approved by Animal Care and Use Ethics Committee of the University of Padova.

## Author Contributions

SR and MR conceived the study. SR, FC, and GS carried out the microbiology experiments. PB and IC conceived and performed the animal trial and the immunology experiments. AA and SR carried out statistical analysis and curated data presentation. AA, MR, and SR wrote the article with contributions from all other authors. All authors contributed to the article and approved the submitted version.

## Conflict of Interest

The authors declare that the research was conducted in the absence of any commercial or financial relationships that could be construed as a potential conflict of interest.

## Publisher’s Note

All claims expressed in this article are solely those of the authors and do not necessarily represent those of their affiliated organizations, or those of the publisher, the editors and the reviewers. Any product that may be evaluated in this article, or claim that may be made by its manufacturer, is not guaranteed or endorsed by the publisher.

## Footnotes

1
http://symprevius.eu
