# Supplementary material for: Phenotypic Traits and Immunomodulatory Properties of Leuconostoc carnosum Isolated From Meat Products
Source: Front Microbiol. 2021 Aug 25;12:730827. doi: 10.3389/fmicb.2021.730827 (PMC8425591; doi:10.3389/fmicb.2021.730827)
Supplement: Supplementary file 1 [file Data_Sheet_1.PDF]

Phenotypic traits and immunomodulatory properties of *Leuconostoc carnosum* strains from meat products

Stefano Raimondi[1], Gloria Spampinato[1], Francesco Candeliere[1], Alberto Amaretti[1,2], Paola Brun[3], Ignazio Castagliuolo[3], Maddalena Rossi[1,2]*

[1]Department of Life Sciences, University of Modena and Reggio Emilia, Modena, Italy,

[2]Biogest-Siteia, University of Modena and Reggio Emilia, Reggio Emilia, Italy

[3]Department of Molecular Medicine, University of Padova, Padova, Italy

*** Correspondence:**
Corresponding Author
maddalena.rossi@unimore.it

Supplementary Material

Supplementary Table 1. Antibodies used in the study

Antigen (host)	Clone	Supplier
CD3 (rat)	17A2	eBioscience (Thermo Fisher Scientific, Waltham, MA, USA)
CD4 (rabbit) Cy7 conjugate	50134-R001	Sino Biological Inc. (Wayne, PA, USA)
CD8 (rat) Cy7 conjugate	5H10	Invitrogen (Thermo Fisher Scientific, Waltham, MA, USA)
CD25	PC61.5	eBioscience
FoxP3	FJK-16s	Invitrogen
CD11c (mouse) Cy7 conjugate	N418	eBioscience
TLR2	D-17	Abcam (Cambridge, UK)
TLR4	HTA125	Santa Cruz Biotechnology (Dallas, TX, USA)
CD80	16-10A1	Immuno Tools
MHC I	FJK-16s	eBioscience

Supplementary Table 2. Table of the phenotypic properties utilized to compute the UPGMA cladogram.

STRAIN	glycerol	erythritol	D-arabinose	L-arabinose	D-ribose	D-xylose	L-xylose	D-adonitol	methyL-β-D-xylopyranoside	D-galactose	D-glucose	D-fructose	D-mannose	L-sorbose	L-rhamnose	dulcitol	inositol	D-mannitol	D-sorbitol	methyL-α-D-mannopyranoside	methyL-α-D-glucopyranoside	N-acetyL-glucosamine	amygdalin	arbutin	esculin	salicin	D-cellobiose	D-maltose	D-lactose	D-melibiose	D-saccharose	D-trehalose	inulin	D-melezitose	D-raffinose
WC0318	0	0	0	0	1	0	0	0	0	0	1	1	1	0	0	0	0	0	0	0	1	1	0	0	1	0	0	0	0	0	1	1	0	0	0
WC0319	0	0	0	0	1	0	0	0	0	0	1	0	1	0	0	0	0	0	0	0	1	1	0	0	1	0	0	0	0	0	1	0	0	0	0
WC0320	0	0	0	0	1	0	0	0	0	0	1	1	1	0	0	0	0	0	0	0	1	1	0	0	1	0	1	1	0	0	1	1	0	0	0
WC0321	0	0	0	0	1	0	0	0	0	0	1	1	1	0	0	0	0	0	0	0	1	1	0	0	1	0	1	0	0	0	1	1	0	0	0
WC0322	0	0	0	0	1	0	0	0	0	0	1	1	1	0	0	0	0	0	0	0	1	0	0	0	1	0	0	0	0	0	1	1	0	0	0
WC0323	0	0	0	0	1	0	0	0	0	0	1	1	1	0	0	0	0	0	0	0	1	1	0	0	1	0	1	1	0	0	1	1	0	0	0
WC0324	0	0	0	0	1	0	0	0	0	0	1	0	1	0	0	0	0	0	0	0	1	1	0	0	1	0	0	0	0	0	1	1	0	0	0
WC0325	0	0	0	0	1	0	0	0	0	0	1	1	0	0	0	0	0	0	0	0	1	1	0	0	1	0	0	0	0	0	1	1	0	0	0
WC0326	0	0	0	0	1	0	0	0	0	0	1	1	1	0	0	0	0	0	0	0	1	1	0	0	1	0	1	1	0	0	1	1	0	0	0
WC0327	0	0	0	0	1	0	0	0	0	0	1	1	1	0	0	0	0	0	0	0	1	1	0	0	1	0	1	1	0	0	1	1	0	0	0
WC0328	0	0	0	0	1	0	0	0	0	0	1	1	1	0	0	0	0	0	0	0	1	1	0	0	1	0	0	1	0	0	1	1	0	0	0
WC0329	0	0	0	0	1	0	0	0	0	0	1	1	0	0	0	0	0	0	0	0	0	0	0	0	1	0	0	0	0	0	1	0	0	0	0

	amidon	glycogen	xylitol	gentiobiose	D-turanose	D-lyxose	D-tagatose	D-fucose	L-fucose	D-arabitol	L-arabitol	potassium gluconate	potassium 2-keto-gluconate	potassium 5-keto-gluconate	citrate	arginine	AMC	AMP	AZM	BAC	CFM	CIP	CLR	KAN	NEO	TET	VAN	Anti-*Listeria*	Biofilm at 30 °C	EPS at 30 °C	GJ survival	IJ survival	Growth at 42°C	Growth with 60g/L NaCl	Growth with 6.25 mg/L H_2O_2
WC0318	0	0	0	0	1	0	0	0	0	0	0	1	0	0	0	0	0	0	0	0	1	1	0	1	0	0	1	0	0	0	0	1	1	1	1
WC0319	0	0	0	0	1	0	0	0	0	0	0	0	0	0	0	0	0	0	0	1	1	1	0	1	1	0	1	0	0	1	0	1	1	0	0
WC0320	0	0	0	1	1	0	0	0	0	0	0	1	0	0	0	0	0	0	0	0	1	1	0	1	1	0	1	0	0	0	0	1	1	1	1
WC0321	0	0	0	1	1	0	0	0	0	0	0	1	0	0	0	0	0	0	0	0	1	1	0	0	0	0	1	1	1	1	0	1	0	0	1
WC0322	0	0	0	1	1	0	0	0	0	0	0	1	0	0	0	0	0	0	0	1	1	1	0	0	0	0	1	0	0	1	0	1	1	0	1
WC0323	0	0	0	1	1	0	0	0	0	0	0	1	0	0	0	0	0	0	0	0	1	1	0	0	0	0	1	1	1	1	0	1	0	0	1
WC0324	0	0	0	0	1	0	0	0	0	0	0	0	0	0	0	0	0	0	0	0	1	1	0	1	1	0	1	0	0	1	0	1	1	0	1
WC0325	0	0	0	0	1	0	0	0	0	0	0	0	0	0	0	0	0	0	0	0	1	1	0	0	0	0	1	0	0	0	0	1	0	1	1
WC0326	0	0	0	1	1	0	0	0	0	0	0	0	0	0	0	0	0	0	0	0	1	1	0	1	1	0	1	0	0	0	0	1	1	1	1
WC0327	0	0	0	1	1	0	0	0	0	0	0	0	0	0	0	0	0	0	0	0	1	1	0	1	1	0	1	0	0	0	0	1	1	0	1
WC0328	0	0	0	0	1	0	0	0	0	0	0	1	0	0	0	0	0	0	0	0	1	1	0	1	1	0	1	0	0	0	0	1	0	0	1
WC0329	0	0	0	0	0	0	0	0	0	0	0	1	0	0	0	0	0	1	0	1	1	1	0	1	0	0	1	0	0	0	0	1	1	0	1

Supplementary Table 3. Resistance at the simulated intestinal juice. Strains were treated for 2 h at 37°C. The residual viability (%) was reported.

Strain	Residual viability
WC0318	100%
WC0319	100%
WC0320	16%
WC0321	100%
WC0322	28%
WC0323	69%
WC0324	100%
WC0325	42%
WC0326	25%
WC0327	20%
WC0328	100%
WC0329	48%

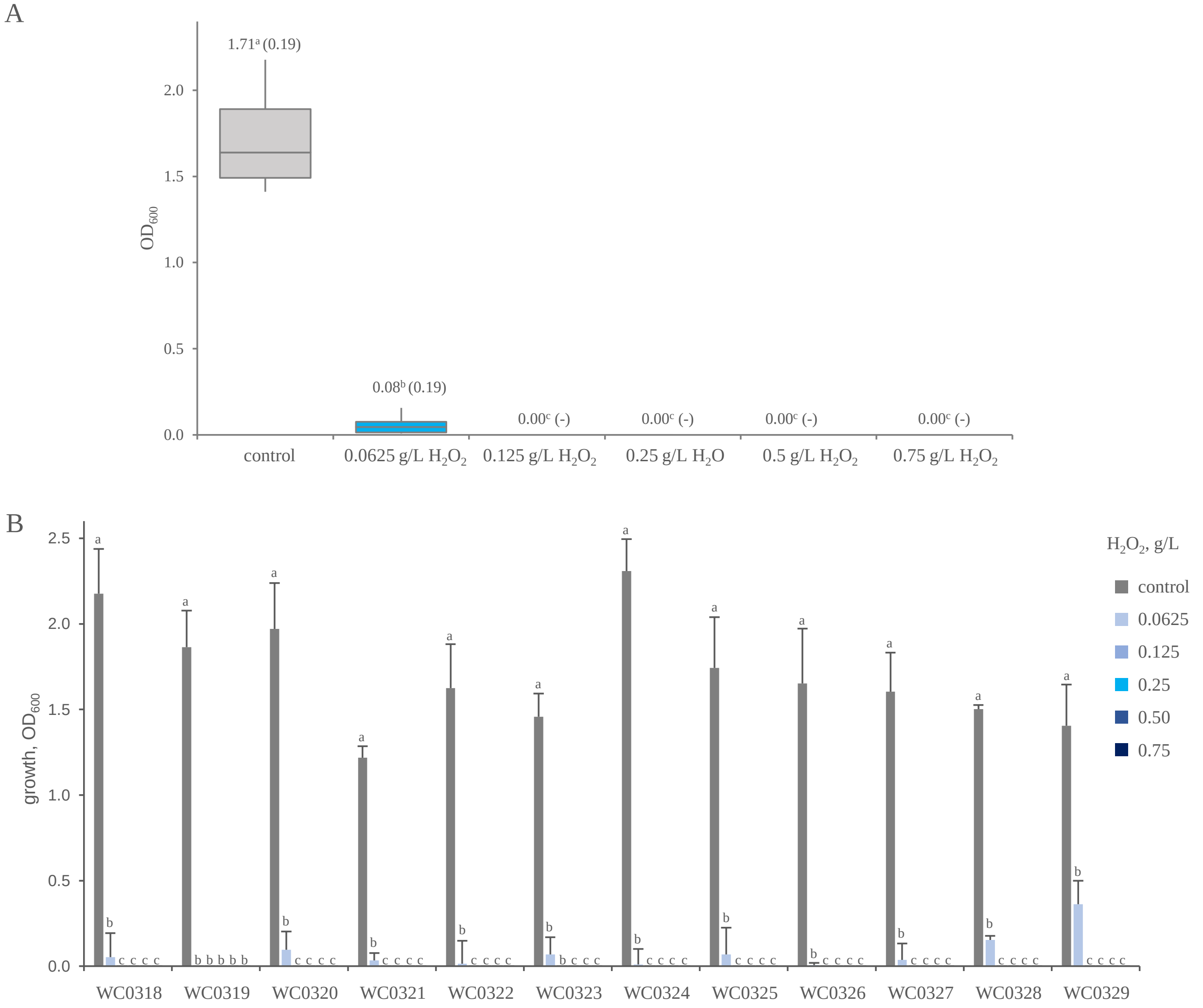

Supplementary Figure 1. Growth of *L. carnosum* strains in presence of increasing concentration of H_2O_2. A) Distribution of the growth yield. Boxes indicate the 25th, 50th, and 75th percentiles; whiskers indicate the 10th and 90th percentiles. The labels report the mean and, in brackets, the coefficient of variation; different letter superscripts indicate means that significantly differ ($P < 0.05$, ANOVA with Tukey's *post hoc*). B) Growth yield of the single strains. Values are means ± SD, n = 3. Within each strain, means with different superscript significantly differed ($P < 0.05$, ANOVA with Tukey's *post hoc*).

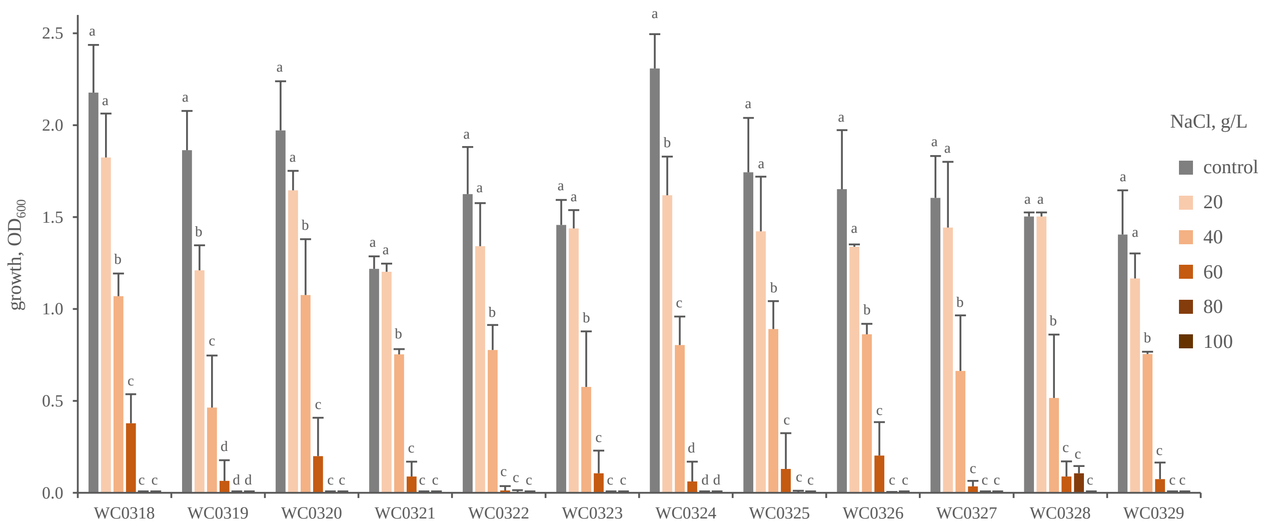

Supplementary Figure 2. Growth of *L. carnosum* strains in presence of increasing concentration of NaCl. Growth yield of the single strains. Values are means ± SD, n = 3. Within each strain, means with different superscript significantly differed ($P < 0.05$, ANOVA with Tukey's *post hoc*).

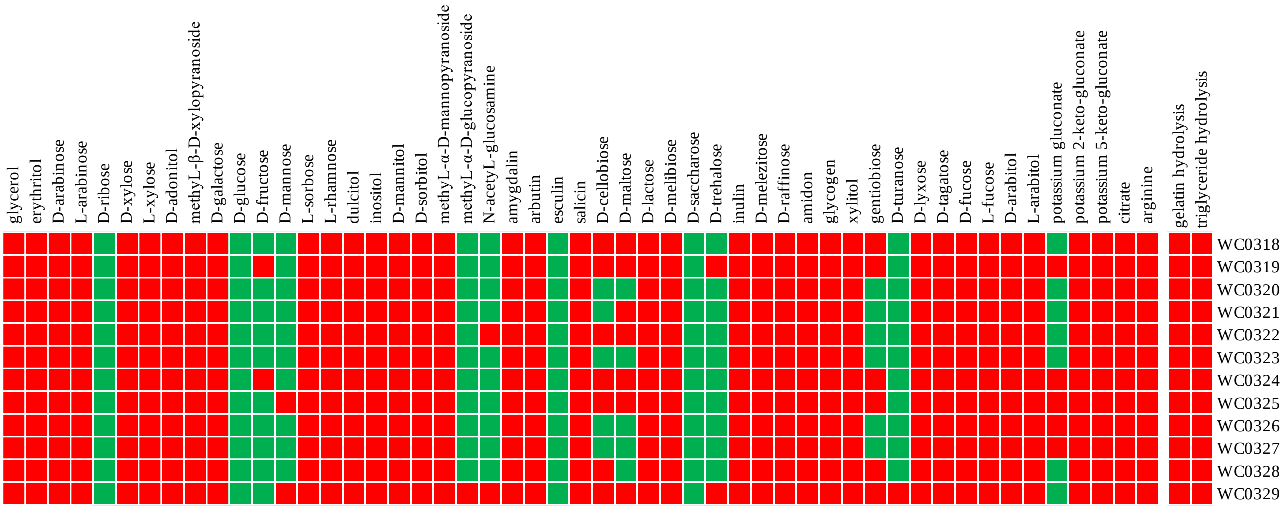

Supplementary Figure 3. Metabolic characterization of 12 *L. carnosum* strains. Utilization of substrates was assayed with API 50 CH, except for arginine and citrate, assayed with specific media. Positive and negative assays are displayed in green and red, respectively.

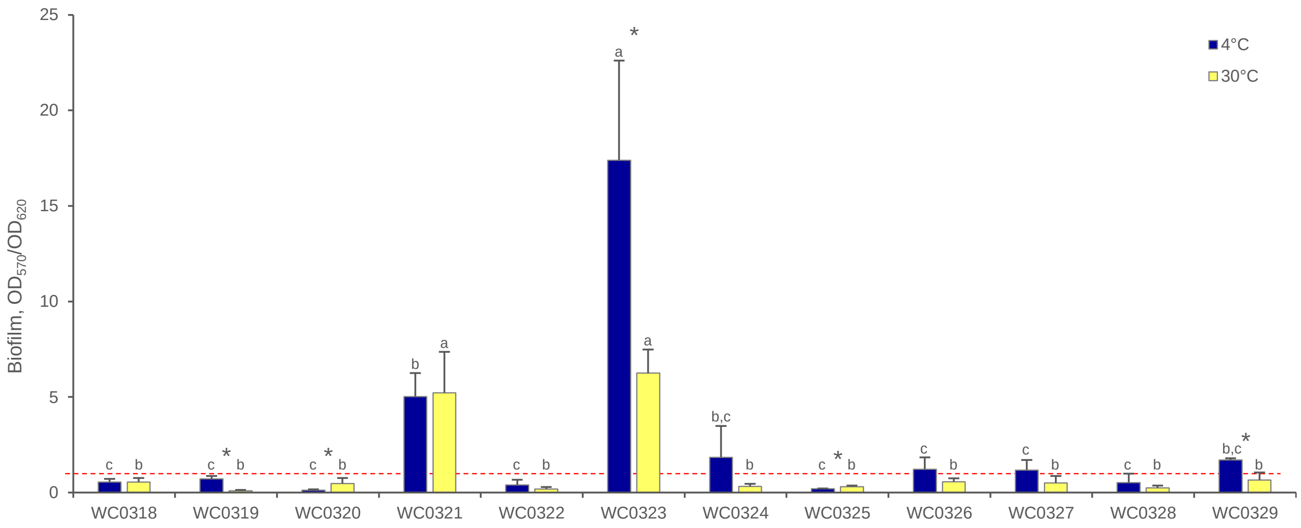

Supplementary Figure 4. Biofilm production in *L. carnosum* strains at 3 and 30 °C, quantified with crystal violet. Values are means ± SD, n = 3. Within each strain, * indicate significant difference between the temperatures ($P < 0.05$, t test). Within temperature, means with different superscript significantly differed ($P < 0.05$, ANOVA with Tukey's *post hoc*). The red dashed line is the threshold of 1.0, above which biofilm formation was considered positive according to Amaretti et al., 2020.
